# Effects of low temperature on growth and metabolism of larval green sturgeon (*Acipenser medirostris*) across early ontogeny

**DOI:** 10.1007/s00360-024-01568-y

**Published:** 2024-07-02

**Authors:** Vanessa K. Lo, Kenneth W. Zillig, Dennis E. Cocherell, Anne E. Todgham, Nann A. Fangue

**Affiliations:** 1 Department of Wildlife, Fish and Conservation Biology, University of California Davis, Davis, CA 95616 USA; 2 Department of Animal Science, University of California Davis, Davis, CA 95616 USA

**Keywords:** Fish, Scaling, Condition, Length–weight relationship

## Abstract

Southern Distinct Population Segment (sDPS) green sturgeon spawn solely in one stretch of the Sacramento River in California. Management of this spawning habitat is complicated by cold water temperature requirements for the conservation of winter-run Chinook salmon. This study assessed whether low incubation and rearing temperatures resulted in carryover effects across embryo to early juvenile life stages on scaling relationships in growth and metabolism in northern DPS green sturgeon used as a proxy for sDPS green sturgeon. Fish were incubated and reared at 11 °C and 15 °C, with a subset experiencing a reciprocal temperature transfer post-hatch, to assess recovery from cold incubation or to simulate a cold-water dam release which would chill rearing larvae. Growth and metabolic rate of embryos and larvae were measured to 118 days post hatch. Reciprocal temperature transfers revealed a greater effect of low temperature exposure during larval rearing rather than during egg incubation. While 11 °C eggs hatched at a smaller length, log-transformed length–weight relationships showed that these differences in developmental trajectory dissipated as individuals achieved juvenile morphology. However, considerable size-at-age differences persisted between rearing temperatures, with 15 °C fish requiring 60 days post-hatch to achieve 1 g in mass, whereas 11 °C fish required 120 days to achieve 1 g, resulting in fish of the same age at the completion of the experiment with a ca. 37-fold difference in weight. Consequently, our study suggests that cold rearing temperatures have far more consequential downstream effects than cold embryo incubation temperatures. Growth delays from 11 °C rearing temperatures would greatly increase the period of vulnerability to predation in larval green sturgeon. The scaling relationship between log-transformed whole-body metabolism and mass exhibited a steeper slope and thus an increased oxygen requirement with size in 11 °C reared fish, potentially indicating an energetically unsustainable situation. Understanding how cold temperatures affect green sturgeon ontogeny is necessary to refine our larval recruitment estimations for this threatened species.

## Introduction

Temperature affects virtually all aspects of physiology in ectotherms including growth and metabolism, which in turn interact in complex ways (Hochachka and Somero 2002). Growth depends on metabolic rate and in return, metabolic rate changes with body size (Moyano et al. 2017). Understanding how temperature affects growth and metabolism provides a baseline for energy budgets and resource allocation that have implications for maximum performance (Schulte 2015), behavior (Killen et al. 2013), and life history strategy (Metcalfe et al. 1995; Killen et al. 2010). For fishes, early life stages are of particular interest, as rates of survival to recruitment are important for population dynamics. Specifically, colder temperatures reduce growth and size-at-age, increasing the duration of a young fish’s vulnerability to predation, and potentially the number of individuals that reach recruitment (Anderson 1988).

Environmental conditions encountered during early life have the potential to affect phenotypic traits later in life—termed carryover effects—and can have implications for performance and fitness (Burggren and Mueller 2015; Saboret and Ingram 2019). Chronic decreases in growth and metabolism due to cold temperatures can impact survival (Wilson et al. 2021), outmigration timing (Gosselin et al. 2021), foraging and swimming ability (Baker et al. 2014), and salinity tolerance (Allen and Cech 2007; Allen et al. 2011). Additionally, these carryover effects can result from relatively short exposures if they occur during critical developmental windows (Mueller et al. 2015).

Green sturgeon (*Acipenser medirostris*) are anadromous, long-lived, and late maturing fish found in the northern Pacific Ocean along the coast of North America (Moyle 2002; Moser et al. 2016). Despite a latitudinally-broad range from Ensenada, Mexico to the Bering Sea off the Alaskan coast, they are considered a species of concern due to the widespread damming of rivers, which has eliminated access to historical spawning areas and altered flow and temperature regimes (Yoshiyama et al. 1998; Moser et al. 2016). Currently, the only known rivers where green sturgeon spawn across their entire distribution are the Rogue River in Oregon (USA), and the Klamath and Sacramento rivers in California (National Marine Fisheries Service 2018). Genetic analyses have shown a strong differentiation between populations spawning in the Rogue and Klamath Rivers, and the Sacramento river, separating the species into a northern (nDPS) and a southern distinct population segment (sDPS), respectively (Israel et al. 2004; Adams et al. 2007). In 2006, sDPS green sturgeon were listed as a threatened species under the Endangered Species Act (ESA) due to population decline, habitat loss and degradation. Additionally, the presence of a single spawning reach in the Sacramento river exposes the sDPS to increased risk of extinction from stochastic events (National Marine Fisheries Service 2018). Therefore, understanding any acute and carryover effects of temperature on green sturgeon early life stages is vital to conservation efforts.

**Fig. 1:**
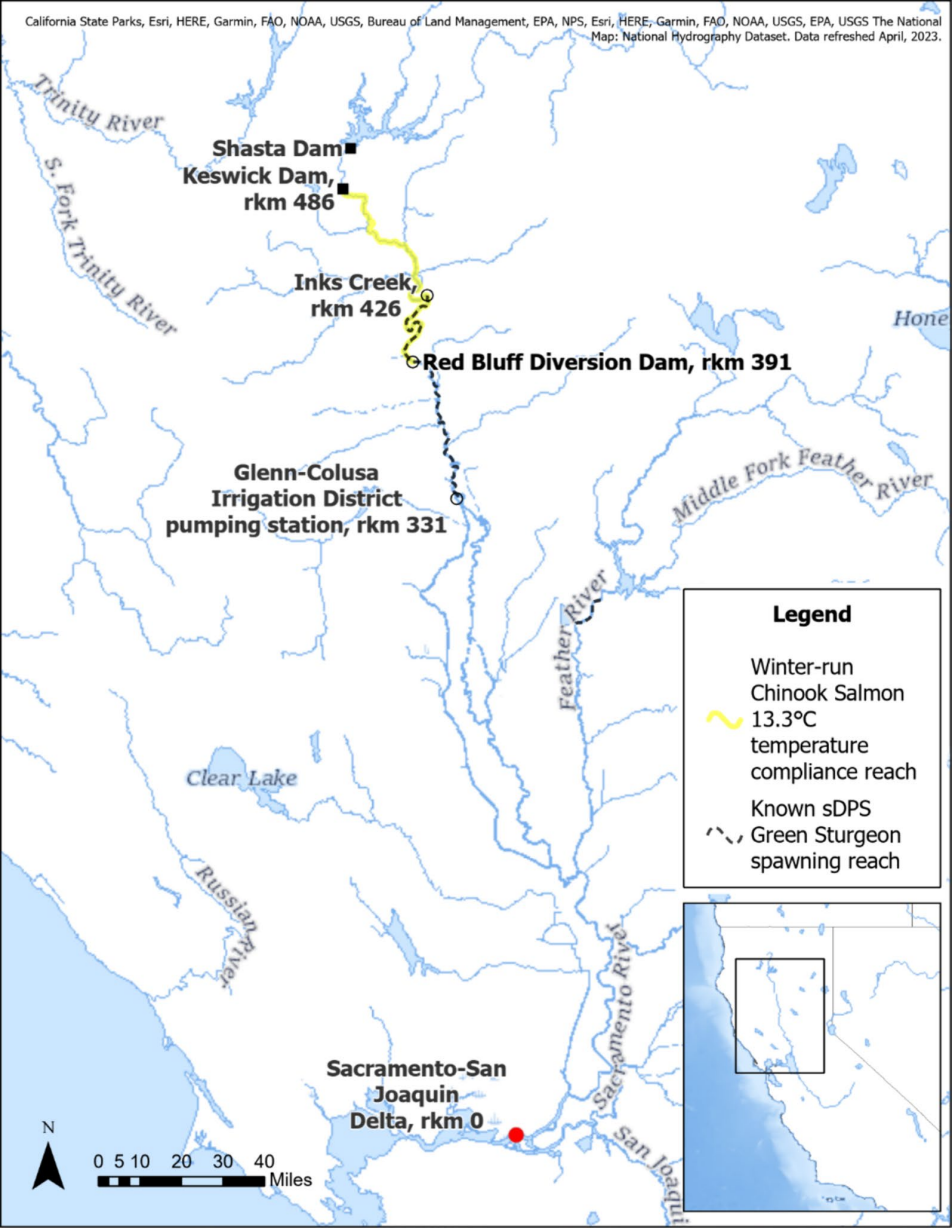
Map of the Sacramento river basin in California, showing the confirmed distribution of sDPS green sturgeon spawning reaches and the winter-run Chinook salmon temperature compliance area. River kilometers (rkm) are calculated as the distance in km upstream from the head of Suisun Bay in the Sacramento-San Joaquin River Delta. Map created using ArcGIS.® software by Esri. Basemap sources: California State Parks, Esri, HERE, Garmin, FAO, NOAA, USGS, Bureau of Land Management, EPA, NPS, National Hydrography Dataset, refreshed April, 2023

Direct evidence of spawning by sDPS green sturgeon, verified using egg mat sampling, has been documented in sequential years within a 94 km stretch of the mainstem Sacramento river from the Glenn-Colusa Irrigation District pumping station at river kilometer (rkm) 331—calculated as the distance upstream from Suisun Bay in the Sacramento-San Joaquin River Delta—to Inks Creek at rkm 426 (Fig. 1; Brown 2007; Poytress et al. 2015; National Marine Fisheries Service 2018). Additionally, in 2011 sDPS green sturgeon were observed to have spawned in the Feather river, a large tributary of the Sacramento river (Seesholtz et al. 2015). Models suggest that in the absence of impassable dams, historical green sturgeon spawning habitat would have included the mainstem Sacramento and San Joaquin rivers, and several major tributaries including the lower Feather, American, and Yuba rivers (Mora et al. 2009). Currently, the mainstem Sacramento river spawning area for green sturgeon is downstream of an adjacent stretch of spawning habitat for endangered winter-run Chinook salmon (*Oncorhynchus tshawytscha*), which historically spawned in now-inaccessible high elevation, cold, spring-fed streams (Lindley et al. 2004). This habitat elimination led to an ESA listing for winter-run Chinook salmon in 1994—earlier than the 2006 listing for sDPS green sturgeon—resulting in management actions that prioritize critical temperature thresholds for winter-run Chinook salmon.

Currently, managers seek to maintain Sacramento river water temperatures below 13.3℃ (56°F) for winter-run Chinook from approximately May to July using cold-water releases from Shasta reservoir (U.S. Environmental Protection Agency 2003). sDPS green sturgeon spawning occurs from April to June, partially overlapping both spatially and temporally with winter-run Chinook salmon (Fig. 1; National Marine Fisheries Service 2018). These temperatures are lower than historic mainstem Sacramento river temperatures and the optimal incubation temperatures of 15–17 °C for nDPS green sturgeon embryos reared in laboratory conditions (California Department of Water Resources 1988; Van Eenennaam et al. 2005). Van Eenennaam et al. (2005) showed that incubation temperatures of 11 °C resulted in decreased hatch rates and shorter hatchlings in nDPS green sturgeon, which are used as a proxy for sDPS green sturgeon due to listing status. Similarly, Poletto et al. (2018) found that juvenile nDPS green sturgeon reared at 11 or 13 °C elicited reduced growth rates compared to fish reared at 16 or 19 °C. Neither of these studies investigated the effects of cold temperature across all early life history stages (i.e., embryo incubation to early juvenile), a data gap we intend to fill with nDPS green sturgeon. Differences in thermal physiology between the two populations remains unknown, given the inability to study early life sDPS in a laboratory, but comparison of age structure and growth from archived sDPS fin rays with age classes ranging from 0 to 26 y suggest similar growth rates to nDPS fish (Ulaski and Quist 2021). Due to the current management temperature threshold, exposing nDPS green sturgeon to temperatures below laboratory-based optimums for growth may more accurately represent the dam-manipulated thermal regime currently experienced by wild sDPS green sturgeon.

The purpose of this study was to assess if incubation temperature results in carryover effects on allometric scaling relationships in both growth and metabolism post-hatch, by using reciprocal temperature transfers after hatch between an optimal and low incubation temperature. Embryos were incubated at an optimal (15 °C) and low (11 °C) temperature until 1 day post-hatch (dph), at which time half of each treatment underwent a reciprocal temperature switch. We predicted that low incubation temperatures would lead to smaller size at hatch and dramatically slow growth rate, reducing size-at-age and leading to differences in length–weight relationships among treatments. Upon the reciprocal temperature switch, we expected to see compensatory growth among fish moved to optimal temperature, continued stunting among fish remaining at the colder temperature, and depression of growth among fish moved from optimal to the colder temperature condition.

## Materials and methods

### Experimental animals

nDPS Green sturgeon embryos were obtained from a broodstock program maintained at the UC Davis Center for Aquatic Biology and Aquaculture’s (CABA) Putah Creek Facility. Green sturgeon adults were originally sourced throughout 1999–2005 from wild Klamath River basin nDPS green sturgeon in collaboration with the Yurok Tribe’s gill-net subsistence fishery and spawned annually (Van Eenennaam et al. 2008). In March 2019, fertile F2 adults (one female and 3 males) were tank spawned at 15 °C following methods described by Van Eenennaam et al. (2012). Injections of gonadotropin-releasing hormone analog and white sturgeon (*A. transmontanus)* pituitary extracts were used to induce spawning, which occurred over a 29 h period. Eggs were hand netted from the tank every 2 h. After tank spawn completion, all three males were checked and confirmed to have spermiated. Therefore, this spawn was composed of three half-sibling families, although the percent hatch contribution from individual males was unknown. The next day, two replicate tanks of approximately 800 eggs each were ramped down to 11 °C ± 0.5 at a rate of 2 °C per day while the other two replicate tanks of 800 eggs were maintained at 15 °C ± 0.5. Embryos were incubated at 15 °C and 11 °C until 1 dph, at which time both replicate tanks per treatment were split evenly. Two of four tanks from each incubation temperature underwent a reciprocal temperature switch, resulting in four total treatments with two tanks each: incubation and rearing at 15 °C, incubation and rearing at 11 °C, incubation at 11 °C and rearing at 15 °C, and incubation at 15 °C and rearing at 11 °C, hereafter listed as 15 °C, 11 °C, 11–15 °C, and 15–11 °C treatments, respectively. The incubation period prior to reciprocal temperature transfers lasted seven days for both 11 °C and 15 °C groups.

Embryos were incubated at 11 °C or 15 °C inside floating mesh baskets within two replicate 350-L temperature-controlled flow-through rearing tanks supplied with aerated water from a dedicated well. Embryos were arranged in a single layer to maximize oxygenation and prevent clumping and the spread of fungus on dead eggs. Dead and fungus covered eggs were removed at least three times per day. Well-water salinity was 0.4 ppt and fish were exposed to natural photoperiod conditions for Davis, CA (38.5°N). Once sturgeon began to hatch and swim freely, they were moved out of the floating mesh baskets and into the tank below. Each treatment was reared in two 470 L replicate tanks for a total of 8 tanks for the remainder of the experiment. Since early developmental stages rely on endogenous yolk reserves (Kamler 2008), fish were not fed until approximately 14 dph, although food was provided at ca.12 dph to orient larvae to chemical cues (Van Eenennaam et al. 2012). Once feeding was detected, larvae were fed ad libitum with semi-moist commercial Starter Crumble feed (Skretting, USA) and excess uneaten feed and feces were removed daily. Feed rates were calculated according to optimal feed rate models for white sturgeon (Deng et al. 2003; Lee et al. 2014) using mean wet mass and water temperature. Feed rates were updated biweekly to account for fish growth. Survival from hatch to exogenous feeding was 71.8% for 15 °C, 91.9% for 11 °C, 85.3% for 11–15 °C, and 70.5% for 15–11 °C treatments. All experimental protocols and fish care were approved by the UC Davis Institutional Animal Care and Use Committee, protocol #21834.

### Growth

Length and weight were collected from 1 to 118 dph after fish were euthanized in a lethal solution of tricaine methanesulfonate (0.5 g L^−1^) buffered with sodium bicarbonate (0.42 g L^−1^) and salt (6.0 g L^−1^). After blotting dry, individuals were laid flat on paper towels and measured to the nearest 0.01 mm using digital calipers (Neiko tools), and 0.0001 g using an analytical balance (Model A-200DS, Denver Instrument Company). Length and weight were collected following metabolic rate (*Ṁ*O_2_) trials (eight fish per trial) and additional data was gathered using random samples of eight fish from each of two replicate treatment tanks. In total, 132, 188, 107, and 165 fish were measured for 15 °C, 11 °C, 11–15 °C, and 15–11 °C treatments, respectively (Table 1).

**Table 1:** Inflection points and slopes presented in length (mm) and mass (g) estimated from the log–log transformed relationship between length and mass in green sturgeon from hatch to 1 g wet mass and 50 mm total length for each temperature treatment

	15 °C		11–15 °C		15–11 °C		11 °C	
	mm	g	mm	g	mm	g	mm	g
First breakpoint								
Total length (mm) and wet mass (g)								
Lower CI (95%)	21.53	0.0575	24.23	0.0692	22.49	0.0583	22.88	0.0592
Estimate	22.45^a^	0.0605	24.92^b^	0.0713	22.97^a^	0.0595	23.39^a^	0.0606
Upper CI (95%)	23.41	0.0637	25.64	0.0735	23.47	0.0607	23.90	0.0620
Second breakpoint								
Total length (mm) and wet mass (g)								
Lower CI (95%)	32.44	0.2237	31.75	0.2119	29.80	0.1581	32.67	0.2294
Estimate	36.16	0.3288	33.10	0.2557	33.16	0.2362	34.70	0.3036
Upper CI (95%)	40.31	0.4835	34.51	0.3085	36.90	0.3528	36.86	0.4018
First segment								
First slope	1.241		1.064		0.961		1.033	
Std. error	0.225		0.442		0.135		0.077	
* n*	25		24		52		71	
Second segment								
Second slope	3.551^c^		4.500^d^		3.757^cd^		4.013^d^	
Std. error	0.101		0.211		0.110		0.097	
* n*	66		25		78		94	
Third segment								
Third slope	2.809		2.816		3.035		2.850	
Std. error	0.028		0.019		0.089		0.116	
* n*	41		58		35		23	

Inflection points are presented with 95% confidence intervals, while slopes are presented with standard errors. Inflection point and slope superscripts indicate statistical significance in pairwise comparisons between treatments. *N* values represent the number of fish included in the regression for each stanza

### Metabolic rate

Oxygen consumption—an indirect measure of aerobic metabolic rate (*Ṁ*O_2_)—of individual embryos was measured using closed-system respirometry in two 24-well microplate systems (Loligo Systems, Denmark) with flow-through water baths supplied separately by 11 °C and 15 °C 350-L temperature-controlled reservoirs. Each 940 µl well contained an optical oxygen sensor spot (PreSens, Germany), which was read using a 24-channel optical fluorescence oxygen reading device and MicroResp™ automated microplate respirometry software (Loligo Systems, Denmark). The microplate system and water bath were kept on an electric rocking platform which gently agitated the contents in the microplate wells to ensure oxygen mixing. Each well’s oxygen sensor spot was calibrated individually with oxygen-free water and fully aerated distilled water at either 11 °C or 15 °C, specific to the following trial. Oxygen-free distilled water was created by adding 1 g sodium sulfite (Na_2_SO_3_) to 100 ml of distilled water. Fully aerated distilled water was created by bubbling ambient air into 100 ml of water for 20 min. Both calibration measurements were conducted while the microplates were inside the water bath to reduce temperature fluctuations. *Ṁ*O_2_ was calculated from the slope of the linear regression fit to each well’s declining O_2_ content during the closed period (10–60 min). Due to the nature of closed respirometry, each embryo produced one *Ṁ*O_2_ measurement, which was only included in analysis if the R^2^ > 0.95. Embryo *Ṁ*O_2_ was measured approximately halfway through development, between 4 and 6 days post fertilization. Experiments were concluded before the oxygen concentration declined below 6.0 mg O_2_ L^−1^—an oxygen concentration that ensures survival and routine metabolism in European sea sturgeon (*A. sturio*) embryos exposed for 48 h (Delage et al. 2020). Background bacterial respiration was assessed by leaving 6 of the 24 wells empty per plate run. Slopes of the 6 blank wells were averaged and subtracted from the remaining 18 wells containing embryos (Svendsen et al. 2016).

*Ṁ*O_2_ of individual larvae was measured by intermittent flow respirometry using an 8-chamber system constructed at University of California, Davis. Each chamber consisted of a glass cylinder with a rubber stopper on each end. Rubber stoppers were pierced with stainless steel tubing fitted with high grade gas impermeable silicone tubing which provided recirculating and flush water flow via two 8-channel low-flow peristaltic pumps (Model BT100-1L, Langer Instruments, USA). To balance rate of oxygen consumption, temperature, and recirculating flow rate, we used two sizes of respirometry chambers (7.77 ± 0.07 mL and 20.78 ± 0.20 mL, mean ± Std Dev) to accommodate the larvae and early juveniles as they grew (Svendsen et al. 2016). Each chamber had an optical oxygen sensor spot (PreSens, Germany) affixed to the inside of the glass chamber wall with silicone glue, which was read through the glass using fiber optic cables and two 4-channel oxygen meters (Witrox 4, Loligo systems, Denmark). The intermittent flow cycle was set such that chambers never fell below 80% O_2_ saturation (8.82 and 8.07 mg O_2_ L^−1^ at 11 °C and 15 °C, respectively) regardless of temperature to ensure the chambers did not become hypoxic (Svendsen et al. 2016). Flush and recirculation periods were controlled using Autoresp™ software (Loligo systems, Denmark). The respirometry chambers and water bath were cleaned and dried daily, and peristaltic pump tubing was bleached, neutralized, and rinsed weekly to prevent bacterial buildup on surfaces. Each respirometry chamber’s sensor spot was calibrated individually with oxygen-free distilled water and fully aerated distilled water every two weeks.

After a 24 h fasting period to eliminate increased postprandial oxygen consumption (Dabrowski et al. 1987), fish were transferred to respirometers and held for ten measurement cycles on average (600–1200 s per measure period), corresponding to roughly 5 h per trial. Preliminary trials showed that two measurement periods were sufficient for fish to recover from handling stress, so the first two measurement periods were removed from analysis. Additionally, data from each trial was visually inspected and measurement periods removed if there were exceedingly variable measurements indicative of equipment abnormalities, such as negative oxygen consumption rate values. Removed measurement periods totaled approximately 7% of all measurement periods. RMR was calculated as the average of the three lowest slopes after any removal of measurement periods (Verhille et al. 2016; Poletto et al. 2017; Zillig et al. 2022). After the trial, sturgeon were euthanized in a lethal solution of tricaine methanesulfonate (0.5 g L^−1^) buffered with sodium bicarbonate (0.42 g L^−1^) and salt (6.0 g L^−1^), blotted dry and laid flat on clean paper towels, then measured to the nearest 0.0001 g and 0.01 mm. Background bacterial respiration was assessed by taking a minimum of three measurement periods after removal of fish, which were averaged and subtracted from the respective chamber’s fish respiration rate. This protocol was completed twice per day, resulting in 16 individual sturgeon *Ṁ*O_2_ assessments per day. *Ṁ*O_2_ is affected by exogenous factors such as temperature as well as endogenous factors such as circadian rhythm, so morning and afternoon *Ṁ*O_2_ measurements were compared within treatments. As morning and afternoon *Ṁ*O_2_ measurements were not significantly different in three of four treatments, they were combined for analysis. Further, our measurements were taken during daylight hours to minimize natural circadian rhythm peaks in *Ṁ*O_2_, as green sturgeon larvae are primarily nocturnal (Kynard et al. 2005). Similarly, Svendsen et al. (2014) found that lake sturgeon (*A. fulvescens*) exhibited peak *Ṁ*O_2_ at dawn with a continued decrease to a metabolic low 3 h after exposure to daylight.

### Data and statistical analysis

**Fig. 2:**
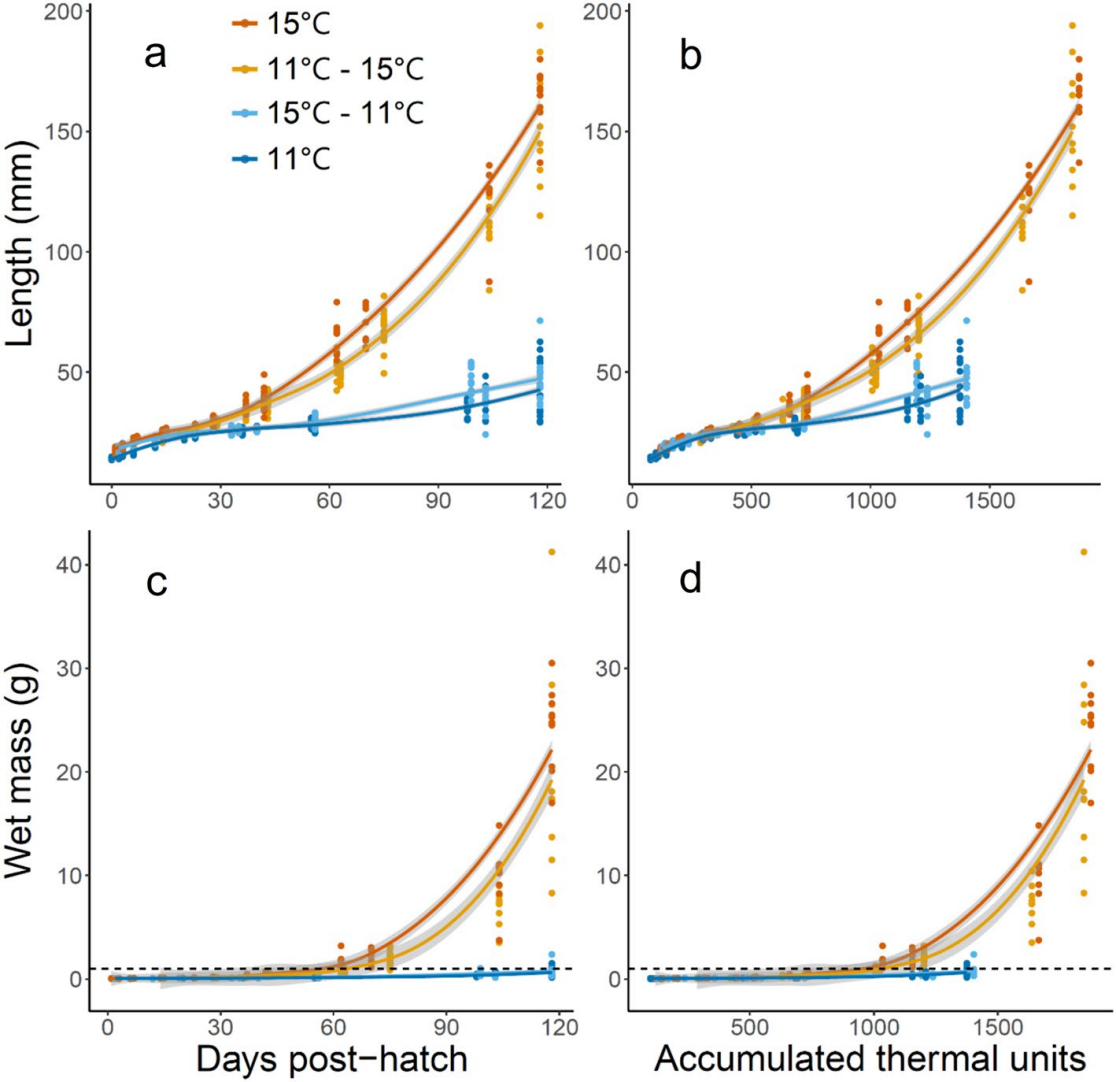
Increase in length and mass of green sturgeon from hatch to 118 days post-hatch by **a**,** c** age and **b**, **d** accumulated thermal units (ATUs) for each treatment. Dark orange points and lines represent larvae incubated and reared at 15 °C (*n* = 132). Light orange points and lines represent larvae incubated at 11 °C and reared at 15 °C (*n* = 107). Light blue points and lines represent larvae incubated at 15 °C and reared at 11 °C (*n* = 165). Dark blue points and lines represent larvae incubated and reared at 11 °C (*n* = 188). Data is smoothed using loess and standard error is indicated in gray

**Fig. 3:**
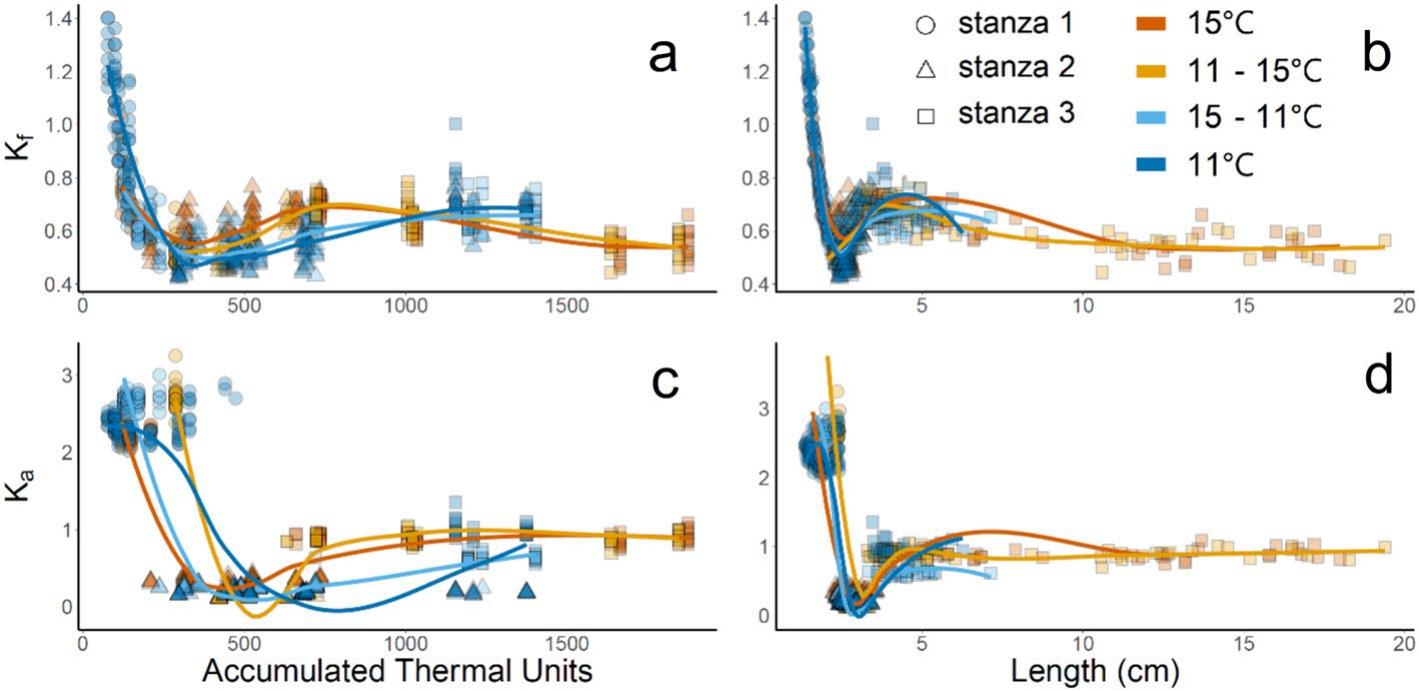
Fulton’s ($K_f$) and Allometric ($K_a$) condition factors by accumulated thermal units (**a**, **c**) and length in cm (**b**, **d**) of prelarval, larval, and early juvenile green sturgeon exposed to different rearing conditions: incubation and rearing at 15 °C (dark orange), incubation at 11 °C and rearing at 15 °C (light orange), incubation at 15 °C and rearing at 11 °C (light blue), and incubation and rearing at 11 °C (dark blue). Lines are fit using Loess smoothing. Shapes indicate which growth stanza a growth measurement falls within each treatment’s LWR, with circles representing stanza 1, triangles representing stanza 2, and squares representing stanza 3

To standardize the effect of temperature over time, accumulated thermal units (ATUs) were calculated to account for differences in temperature exposure. One thermal unit is defined as one degree Celsius experienced in a 24-h period. Thus an embryo incubated at 15℃ would accumulate 15 thermal units per day (Boyd et al. 2010). Accumulation of ATUs commenced once embryo final acclimation temperatures were reached. ATUs were calculated for the duration of the experiment and used in addition to age (dph) to visualize growth (Fig. 2) and condition factor (Fig. 3) over time.

**Fig. 4:**
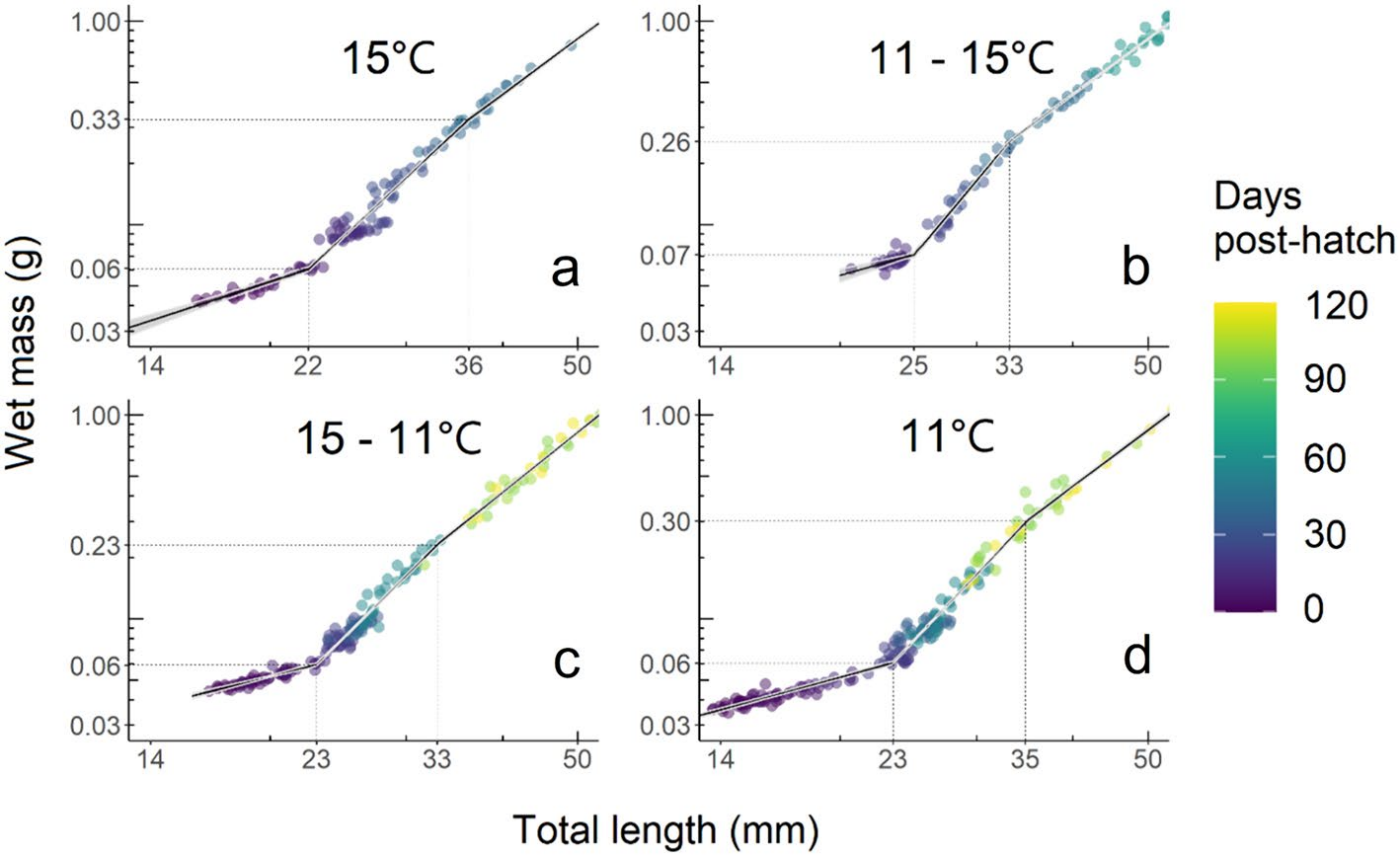
Length–weight relationships of green sturgeon larvae **a** incubated and reared at 15 °C, **b** incubated at 11 °C and reared at 15 °C, **c** incubated at 15 °C and reared at 11 °C, and **d** incubated and reared at 11 °C plotted on log–log transformed axes. Piecewise linear regression estimated using the *segmented* package in R revealed three growth stanzas separated by two inflection points in all treatments. Each stanza is fit to the equation $\log (length) = \log(a) + b* \log (mass)$, where length is in millimeters and mass is in grams. Colored points represent raw data with age shown by color gradient, lines represent the predicted linear regression, and gray ribbons represent standard error of the fit. Inflection points are indicated by dotted vertical and horizontal lines indicating associated lengths and weights at the axes

Length–weight relationships were compared among treatments by log transforming both wet body mass and total length, then fit with piecewise linear regression using the *segmented* package (Muggeo 2003, 2008). The Davies’ test from the *segmented* package was used to analyze each treatment for a non-zero difference-in-slope parameter, providing an estimate of two statistically significant inflection points. Linear models were iteratively fit to identify the two inflection point locations and confidence intervals, as well as regression parameters for the three linear segments separated by the inflection points, hereafter referred to as growth stanzas (Table 1, Fig. 4). Inflection points were compared in a pairwise fashion and considered significant by assessing whether the confidence interval for the difference in means did not include zero. Each of the three individual stanza slopes were compared between treatments by pairwise comparison using the Tukey method in the R package *emmeans*. Results were considered significant at *p* < 0.05.

Condition factor was calculated two ways for each length and weight measurement for additional comparison between treatments. Fulton’s condition factor ($K_f$) was calculated using the equation $K_f = 100\frac{W}{L^3 }$ where *W* = wet mass in grams and *L* = length in cm, assuming isometric growth represented by a coefficient of 3 derived from the slope of length–weight relationships (LWRs) in many fish species (Froese 2006). Allometric condition factor ($K_a$) was calculated using the equation $K_a = 100\frac{W}{L^b }$, where *W* = wet mass in grams, *L* = length in cm, and *b* is the slope of the LWR for each treatment and growth stanza (Cone 1989). $K_a$ allows comparison of condition factor while preserving differences between growth stanzas.

*Ṁ*O_2_ was recorded using MicroResp™ and AutoResp™ software for embryos and larvae, respectively (Loligo Systems, Denmark), and data analyses performed using R (version 4.2.1). Differences between mass-specific *Ṁ*O_2_ of embryos incubated at 11 °C and 15 °C were assessed using T-tests. For larvae, whole-body *Ṁ*O_2_ and wet body mass were log transformed and tested in *segmented* for any potential inflection points. As none were found, data for each treatment were fit to the equation $\log( {\dot{M}} O_{2 }) = \log \, (a) + b *\log( x)$, where ${\dot{M}} O_{2 }$ is in mg O_2_ individual^−1^ h^−1^, and $x$ is wet mass in grams. Slopes compared by pairwise comparison using the Tukey method in the R package *emmeans.* Slopes from the first growth stanza were compared to the expected value of 1 for larval fishes using T-tests (Bochdansky and Leggett 2001).

## Results

Both 11 and 15 °C incubation temperatures hatched within 24 h after an 8 d incubation, but differed in size at hatch. For 11 and 15 °C incubation temperatures, respectively, length was 14.33 ± 0.17 mm and 17.52 ± 0.30 mm (mean ± std. err), weight was 0.0374 ± 0.0004 g and 0.0441 ± 0.0005 g (mean ± std. err), and both were significantly different between treatments (both *p* < 0.0001). As fish aged, measurements of length and weight to 118 dph revealed that fish reared at 11℃ (11℃ and 15–11 °C treatments) had dramatically slowed growth, resulting in large differences in size-at-age. For example, the age at which fish achieved 1 g wet mass was ~ 60 dph for fish reared at 15 °C reared fish and ~ 120 dph for fish reared at 11 °C (dashed horizontal line, Fig. 2c). Differences in size-at-ATU also persisted between rearing temperatures, indicating that the sole effect of mean temperature on early life stages are insufficient to explain developmental trends (Fig. 2b, d).

Log–log transformed wet body mass and total length spanning prelarval, larval, and early juvenile stages (to 118 dph) exhibited three distinct growth stanzas and two inflection points (Fig. 4, Table 1). At the first inflection point, fish length among treatments was significantly larger only for the 11–15 °C treatment, while fish length at the second inflection point was not significantly different among any of the treatments. Each of the three stanzas were fit to the equation $\log( length) = \log (a) + b* \log( mass)$, where *length* is in mm and *mass* is in g. Among treatments pairwise comparisons of first stanza slopes were not significantly different, neither were any pairwise comparisons of third stanza slopes. However, the second stanza of the 15 °C group was significantly different from the 11 °C group (*p* = 0.007) and the 11–15 °C group (*p* = 0.046) (Table 1). Altogether, there was very little difference in developmental trajectories with respect to size at developmental transitions, but an increasingly massive size-at-age difference between the 15 °C and 11 °C rearing temperatures persisted.

Condition factor varied widely depending on both the method of calculation ($$K_f$$ vs. $$K_a$$) and growth stanza, although patterns were similar (Fig. 3). Fish exhibited the highest $$K_f$$ and $$K_a$$ values during prelarval endogenous stages (first growth stanza), lowest values during larval stage (second growth stanza), and moderate values after transition to early juvenile morphology (third growth stanza). Due to the fixed growth coefficient of 3, $$K_f$$ was continuous through growth, while $$K_a$$ was grouped by stanza. $$K_f$$ ranged from 0.42 to 1.40, while $$K_a$$ ranged from 0.11 to 3.25. It must be noted that the condition factor values ($$K_f$$ or $$K_a )$$ can only be compared within life stage and method of calculation.

**Fig. 5:**
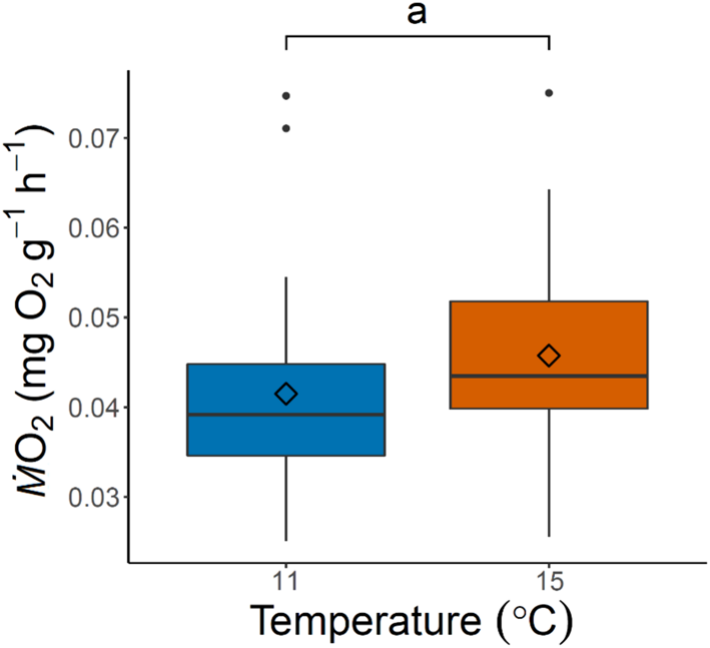
Mass-specific metabolic rate (mg O_2_ g^−1^ h^−1^) of embryos incubated at 11 °C and 15 °C, shown in blue and orange, respectively. The center line of the boxplots represents the median, the box represents the inter-quartile range (IQR), the whiskers extend 1.5 times IQR, and diamonds represent the mean. Mass-specific metabolic rate was not significantly different (*p* = 0.1417) between the two incubation temperatures

**Fig. 6:**
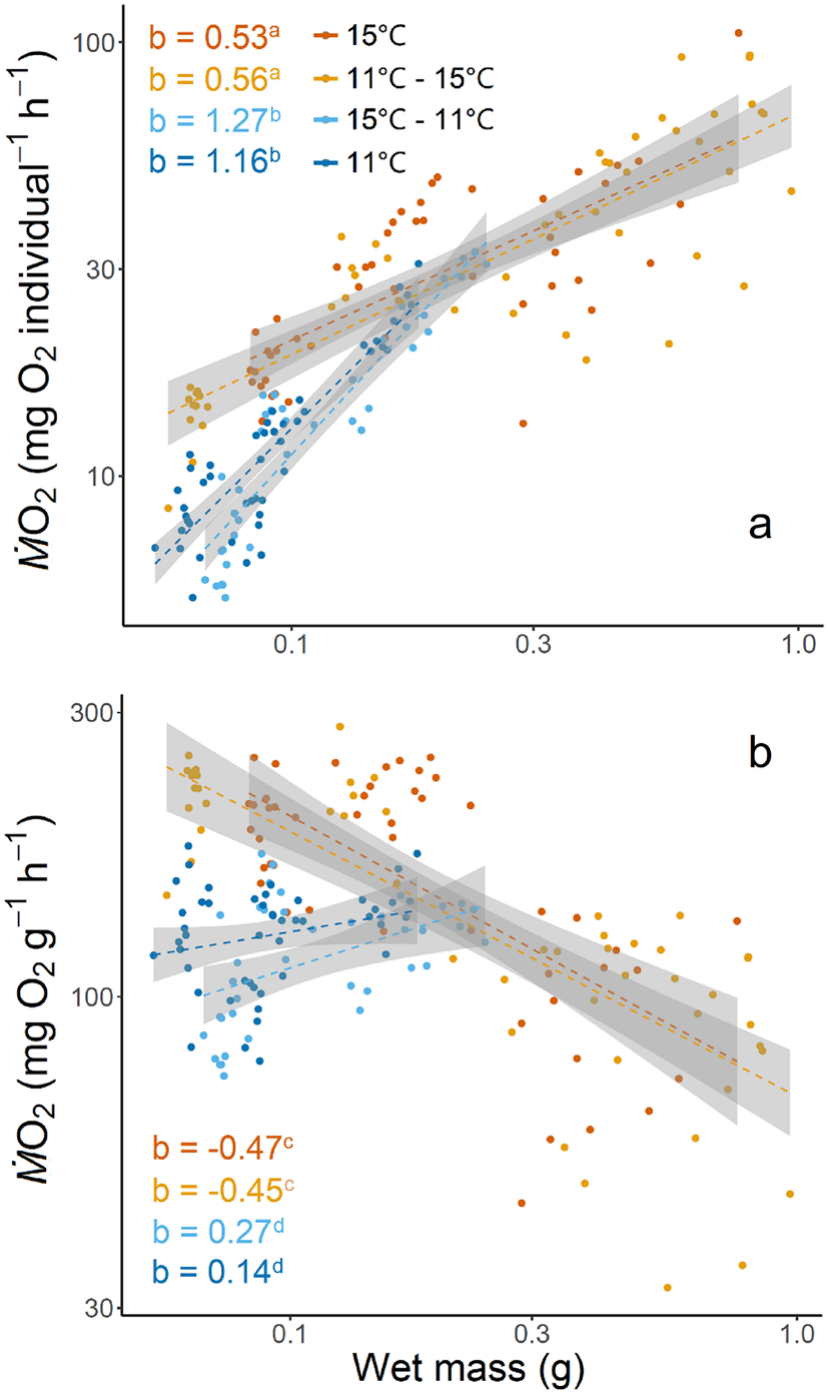
Whole-individual metabolic rate (**a**: mg O_2_ individual^−1^ h^−1^) and mass specific metabolic rate (**b**: mg O_2_ g^−1^ h^−1^) against fish wet mass (g) of green sturgeon through ontogeny to 63 dph, reared at 11 °C or 15 °C, plotted on log–log transformed axes. Relationships were fit using the equation $\log ({\dot{M}} O_{2 }) = \log (a) + b *\log (x)$, where $x$ is wet mass in g. For whole-individual metabolic rates (**a**) dark orange points and line represent larvae incubated and reared at 15 °C, described by $\log( {\dot{M}} O_{2 } )= \log (1.84) + 0.53* \log (x)$. Light orange points and line represent larvae incubated at 11 °C and reared at 15 °C, described by $\log( {\dot{M} } O_{2 } )= \log (1.84) + 0.56* \log (x)$. Light blue points and line represent larvae incubated at 15 °C and reared at 11 °C, described by $\log( {\dot{M}} O_{2 } )= \log (2.32) + 1.27* \log (x)$. Dark blue points and line represent larvae incubated and reared at 11 °C, described by $\log( {\dot{M} } O_{2 } )= \log (2.27) + 1.16* \log (x)$. For mass-specific metabolic rates (**b**) dark orange points and line represent larvae incubated and reared at 15 °C, described by $\log( {\dot{M} } O_{2 } )= \log (1.84) - 0.20 *\log (x)$. Light orange points and line represent larvae incubated at 11℃ and reared at 15 °C, described by $\log( {\dot{M} } O_{2 }) = \log (1.83) + 0.19* \log (x)$. Light blue points and line represent larvae incubated at 15 °C and reared at 11 °C, described by $\log( {\dot{M} } O_{2 } )= \log( 2.32) + 0.12* \log( x)$. Dark blue points and line represent larvae incubated and reared at 11 °C, described by $\log( {\dot{M} } O_{2 } )= \log (2.25) + 0.06 *\log (x)$. Slope values, *b*, for each treatment are indicated in each treatment’s respective color with letters indicating significance between values via pairwise comparison

Mass specific *Ṁ*O_2_ of embryos incubated at 11 °C and 15 °C were not significantly different, although values were lower at 11 °C as expected (*p* = 0.1417, Fig. 5). Slopes for log-transformed whole individual *Ṁ*O_2_ by weight of larvae were all significant (*p* < 0.0001) and described by the following relationships: 15 °C treatment fish were described by $\log( {\dot{M}} O_{2 } )= \log( 1.84) + 0.53* \log (x)$; 11–15 °C treatment fish were described by $\log( {\dot{M}} O_{2 }) = \log (1.84) + 0.56*\log (x)$; 15–11 °C treatment fish were described by $\log( {\dot{M} } O_{2 }) = \log( 2.32) + 1.27* \log (x)$; 11 °C treatment fish were described by $\log ({\dot{M}} O_{2 } )= \log (2.27) + 1.16*\log (x)$ (Fig. 6a). Slopes for log-transformed mass specific *Ṁ*O_2_ by weight of larvae were significant (*p* < 0.001) for all but the 11 °C treatment (*p* = 0.1) and are described by the following relationships: 15 °C treatment fish were described by $\log( {\dot{M} } O_{2 } )= \log (1.84) - 0.47*\log (x)$; 11–15 °C treatment fish were described by $\log ({\dot{M}} O_{2 } )= \log (1.84) - 0.46*\log (x)$; 15–11 °C treatment fish were described by $\log( {\dot{M}} O_{2 }) = \log (2.32)+ 0.27*\log (x)$; 11 °C treatment fish were described by $\log ({\dot{M} } O_{2 } )= \log (2.25) + 0.14*\log (x)$ (Fig. 6b).

Pairwise comparisons of treatment slopes showed significant differences (*p* < 0.0001) between all treatments except those that shared rearing temperatures: 15 °C and 11–15 °C (*p* = 0.98) and 11 °C and 15–11 °C (*p* = 0.90). When the first stanzas’ slopes were compared to the value of *b* = 1, all treatments were significantly different (15 °C, *p* < 0.0001; 11–15 °C, *p* < 0.0001; 15–11 °C, *p* = 0.0149) except the 11 °C treatment (*p* = 0.1890).

## Discussion

### Growth

For nDPS green sturgeon, exposure to low temperatures during early life development post-hatch appears far more consequential than exposure during egg incubation. Length at hatch was greater in fish incubated at 15 °C, but growth trajectories showed this difference subsiding over time. In contrast, 11 °C rearing temperatures caused significant stunting and large differences in size-at-age despite similar growth trajectories (Fig. 2a, c). This growth delay during early life development is not explained entirely by temperature exposure. Although there is some consistency in development at ATU in the very earliest stages between 500 and 700 ATU depending on length or weight, rearing treatments eventually diverged by temperature (Fig. 2b, d). The insufficiency of a simplifying model such as ATUs is not surprising, but the similarity in the earliest stages supports evidence in brook charr (*Salvelinus fontinalis*) indicating an embryo-alevin (i.e., post-yolk resorption) boundary where maternal genetic effects change to those of the individual organism (Perry et al. 2004).

All fish hatched within a 24 h period despite the estimated post-fertilization to peak hatch times of ~ 150 h for incubation at 15 °C and ~ 220 h for incubation at 11 °C (Van Eenennaam et al. 2005). However, any differences in hatch time caused by temperature were likely obscured by the 29 h range of fertilization times and the 48 h initial temperature ramp at a rate of 2 °C per day from the spawning temperature of 15 °C to the incubation temperature of 11 °C. Our fish hatch lengths are also consistent with findings from Van Eenennaam et al. (2005), though they additionally found that dry weights did not differ between treatments. In shortnose sturgeon (*A. brevirostrum*) and Atlantic sturgeon (*A. oxyrinchus*), post-hatch larvae reared at lower than optimal temperatures exhibited slower yolk-sac utilization rates, but temperature did not affect size at completion of yolk absorption (Hardy and Litvak 2004). Interestingly, yolk utilization efficiency was independent of temperature in both species, suggesting that sturgeon are capable of balancing the increasing metabolic requirement of warmer temperatures with a reduction in developmental time and vice versa when in colder temperatures.

After hatch, 11 °C rearing severely delayed green sturgeon larvae growth, especially during the larval and early juvenile stages (second growth stanza and second inflection point, Fig. 4), at which rearing temperature caused fish to diverge in size-at-age (Fig. 2). Our study shows that rearing at 11 °C would greatly increase the period of vulnerability to predation in the wild, given that many native and non-native piscivorous fish in the Sacramento river are gape limited (Baird et al. 2020). In shovelnose sturgeon, simulation experiments with long-term environmental data suggest that slow growth and low survival of larval shovelnose sturgeon likely play a large role in recruitment failure (Goto et al. 2015). Stunted growth also has cascading effects on foraging ability, swimming ability, and osmoregulation, all of which could contribute to poor recruitment. For example, salinity tolerance in nDPS green sturgeon increases linearly with body length and weight (Allen et al. 2011), in conjunction with a rapid increase in mitochondria-rich cell size in the gills between 15 and 45 cm total length (Allen et al. 2009). Similarly, nDPS green sturgeon critical swimming velocity increases with size in larval fish aged 20 to 60 dph (Verhille et al. 2014). While the predicted effects of climate change are expected to increase water temperatures on average, it is still important to consider the effects of managed artificially cool water on sDPS green sturgeon, especially in spawning habitats overlapping spatially and temporally with winter-run Chinook salmon (Fig. 1).

Log-transformed LWRs revealed three similar developmental stages, despite the considerable differences in size-at-age among treatments (Fig. 4). The three growth stanzas correspond to prelarval, larval, and early juvenile stages and the two inflection points represent the transition between developmental periods (Table 1). These developmental changes likely have survival and recruitment implications as changes in form reflect changes in function and habitat usage (Fuiman and Werger 2002). Changes in slope across stanzas are known to be influenced by both endogenous and exogenous factors such as developmental stages, temperature, or starvation (Froese 2006). In total, our treatments exhibited minimal differences in slope (indicative of growth trajectory) and inflection points (indicative of size at transition between developmental stages), despite severely slowed growth in both 11 °C reared treatments. Total lengths corresponding to the first inflection point were significantly different in only the 11–15 °C treatment. By the second inflection point, however, none of the total lengths were significantly different among treatments. Additionally, slopes were not significantly different among treatments within the first and third stanzas, respectively, and only two pairwise treatments in the second stanza had significantly different slopes (Table 1). Thus, any differences in hatch length resulting from incubation temperature appeared to have dissipated by the second inflection point, supporting Hardy and Litvak’s (2004) observation in shortnose and Atlantic sturgeon that yolk utilization efficiencies were independent of temperature, with fish attaining identical sizes at completion of yolk absorption.

While growth stanzas in larval green sturgeon are not a new discovery, they have not been assessed at low temperatures. Gisbert and Doroshov (2006) reared fish up to 50 dph at 16 °C and found three stanzas in their LWRs with slopes of 0.76, 2.35, and 3.38 and inflections at 20 and 25 mm. Interestingly, the slopes became steeper with size and both inflection points occurred close to the size at which our first inflection points occurred (23–25 mm). The limited size range (10–45 mm) observed in Gisbert and Doroshov (2006) could explain this discrepancy. To replicate their analysis, we limited our data to 50 dph and repeated the analysis. We found only one inflection point for all treatments except 11–15 °C, which exhibited two inflection points and slope steepness patterns very similar to the original data of our study. As our full dataset substantially expands the range of sizes studied for 15 °C rearing (14–200 mm) it is possible that differences in the scale of data would affect the fitting of piecewise linear regression. Additionally, our fish originated from one mother with three fathers and it has been shown that maternal effects can exert a strong influence on very early life developmental trajectories, which subside over time to paternal influence (Lindholm et al. 2006). We suspect the minimal differences in length at the first inflection point among treatments despite a smaller hatch size in 11 °C incubated eggs could be a result of maternal effects. Differences in developmental trajectories across embryos from different mothers is a future avenue for study.

As expected, due to the similarities in LWR across treatments, patterns for $K_f$ and $K_a$ were similar across treatments when comparing fish of the same length (Fig. 3b, d). When comparing by ATU, the pattern was delayed in cold-reared fish, showing a lag in development associated with rearing at 11 °C (Fig. 3a, c). Kappenman et al. (2009) measured $K_f$ values of ~ 0.28 to 0.33 in juvenile shovelnose sturgeon (*Scaphirhynchus platorynchus*) reared to 87 dph at temperatures from 8 to 30 °C, with optimal $K_f$ values between 16 and 20 °C. Ultimately, standalone condition factor metrics from wild green sturgeon of these life stages are not likely to be informative due to the size-at-age differences and lack of thermal history information.

Overall, reduced size-at-age observed in cold-reared fish and similar LWR and condition factors suggest that length measurements—a widely-measured, non-lethal metric of field-based sDPS green sturgeon growth—cannot be correlated accurately to age without prior knowledge of a fish’s temperature history. Importantly, length measurements are the only individual-based data collected for sDPS green sturgeon in the Sacramento river, because they are captured incidentally by rotary screw traps optimized for and targeting outmigrating salmonid smolts. Length measurements are then compared to optimal growth charts to back calculate age and estimated spawning dates. Furthermore, these incidental captures are the primary source of information on wild larval sDPS green sturgeon habitat utilization, habitat type, and timing of downstream migration. For instance, summaries of sDPS green sturgeon incidental captures used to assess relative abundance and species trends exhibited median lengths of 29 mm (between 1994 and 2000) and 27.3 mm (between 2002 and 2012) (Gaines and Martin 2001; Poytress et al. 2015). Both sizes fall within the second stanza of larval sturgeon growth (Fig. 4), which was the most variable across treatments, and would lead to age estimates of 23 to 56 dph in our treatments, highlighting the inherent uncertainty of length-to-age predictions. We suggest measuring weight along with length for all incidental captures of green sturgeon juveniles in the Sacramento river to better understand how fish are developing in this watershed, and to build a dataset of LWRs across years and environmental conditions for comparison.

### Metabolic rates

To our knowledge, this study is the first to report metabolic rates for green sturgeon embryos. Given the influence of temperature on ectotherms, embryos followed expected trends with lower metabolic rates at 11 °C compared to 15 °C, though they were not significantly different (Fig. 5; Hochachka & Somero 2002; Fry 1971). *Ṁ*O_2_ values reported from the log–log relationship between larvae and wet mass are assumed to represent routine metabolic rate rather than standard metabolic rate, and are presented as whole-individual (Fig. 6a) and mass-specific values (Fig. 6b) (Chabot et al. 2016; Peck and Moyano 2016). Although larvae were separated from food prior to experiments, confined within the respirometry chambers, and measured during low activity daylight hours, our measurements inevitably included some metabolic costs of growth and activity, as larvae were never completely quiescent regardless of acclimation duration during preliminary trials (Kynard et al. 2005; Svendsen et al. 2014).

The slope, *b*, of the relationship between whole-individual *Ṁ*O_2_ and wet mass are used to explain the metabolic scaling of fish. Attempts have been made to describe a universal value of *b* for fishes, but the literature to date for larvae has focused on marine species. These studies suggest that juveniles and adults scale with exponents close to 0.8 or 0.9, while larval fish scale isometrically with an exponent of 1 due to organogenesis, rapid development, and locomotion costs (Giguère et al. 1988; Clarke and Johnston 1999; Glazier 2005; Jerde et al. 2019). However, within individual species much more variation is typically seen, showing bi- or tri-phasic relationships in metabolic scaling (Glazier 2005). Consequently, the broader scaling relationships are often only evident where the data spans 6 or 7 orders of magnitude in mass and such datasets are uncommon (Post and Lee 1996; Bochdansky and Leggett 2001).

Temperature influenced how whole-individual *Ṁ*O_2_ scaled with wet mass across prelarval to early juvenile stages of green sturgeon (Fig. 6a). Treatments sharing 11 °C rearing temperatures (11–11 °C and 15–11 °C) exhibited steeper mass scaling exponents of 1.16 and 1.27, respectively, while those sharing 15 °C rearing temperatures (11–15 °C and 15–15 °C) had exponents of 0.56 and 0.53, respectively (*b* values in Fig. 6a). Our *b* values are between or lower than the exponents given for green sturgeon attributed to exogenous and endogenous feeding stages (1.04 and 1.64, respectively) reared at 16 °C (Gisbert et al. 2001), and within the range of 0.42–1.54 observed in 6 to 24 dph white sturgeon reared at 14 °C in either gravel substrate or no substrate (Boucher et al. 2018). Larger *b* values indicate that total metabolism increases more quickly per unit mass, while a *b* of 1 indicates isometric metabolic scaling with mass. High mass exponents (i.e., *b*) in early life teleosts are widespread but not universal, with exponents greater than 1.0 more common in pre-feeding larvae (Rombough 1988). The larger *b* values in our 11 °C reared fish relative to 15 °C suggest that these baseline functions and growth at suboptimal low temperature is more energetically costly (Fig. 6a).

The location of the inflection points in metabolic scaling appear to vary dramatically among species and are hypothesized to correlate to ontogenetic changes in the mass scaling of respiratory surfaces (e.g., cutaneous surfaces and gill lamellar surface area) and not with size at metamorphosis (Post and Lee 1996). We did not detect any statistically significant inflection points in our *Ṁ*O_2_ relationships, which did span metamorphosis from prelarval to larval (first inflection point, Fig. 4) and larval to early juvenile stages (second inflection point, Fig. 4). When compared to the expected marine larval exponent of 1, all treatments except 11 °C were significantly different (Fig. 6a), possibly due to the large size of larval green sturgeon relative to that of many marine larval fishes to which this trend seems to apply (Giguère et al. 1988).

Our mass-specific *Ṁ*O_2_ values suggest that cold rearing temperatures are more energetically costly and may contribute to dramatically slowed growth, as evidenced by the positive relationship of mass-specific *Ṁ*O_2_ with wet mass in cold-reared fish (Fig. 6b). Typically, a decrease in mass-specific metabolic rate is expected with an increase in mass and is believed to be related to the increase in the ratio of low metabolic activity tissues to high metabolic activity tissues (e.g., organs) (Oikawa et al. 1991). The lower absolute values for *Ṁ*O_2_ in 11 °C reared fish relative to 15 °C reared fish are expected to compromise the ability of fish to capture prey and avoid predation (Wuenschel et al. 2004; Killen et al. 2007).

### Ecological consequences

Early life stages for many species of fish often suffer high mortality rates, which can limit recruitment (Bailey and Houde 1989; Houde 2008). Thus, fast growth and larger size improve survival potential, and sturgeon are no exception (Anderson 1988). While vulnerability to predation in the wild depends on many factors such as morphology, behavior, distribution patterns, and environmental conditions, laboratory predation studies indicate that young white sturgeon < 50 mm are readily consumed by benthic predator species such as channel catfish (*Ictalurus punctatus*), and prickly sculpins (*Cottus asper*) (Gadomski and Parsley 2005). Pelagic predators such as striped bass (*Morone saxatilis*) and largemouth bass (*Micropterus salmoides*) prefer other soft bodied prey, but will still consume or attempt to consume green sturgeon (Baird et al. 2020). Waraniak et al. (2018) used molecular diet analysis to detect lake sturgeon DNA in diet contents from 16 of 28 predator species examined, finding that most predators preyed on larval lake sturgeon at similar rates. They also found that greater proportions of alternative prey and higher discharge rates, which covaries with turbidity, decreased predation rates on larval sturgeon, while increased lunar illumination increased predation rates. There has been a 50% decrease during the last 50 years in sediment load in the Sacramento river due to dams trapping sediment behind reservoirs, armoring of river channels, and deposition of sediment in flood bypasses (Stern et al. 2016). As turbidity is related to sediment, this reduction in sediment discharge could be a contributing factor to sDPS green sturgeon population decline if predation is an important source of mortality.

Our study showed that attaining a somewhat protective size of 50 mm took 120 dph at 11 °C, which was roughly twice as long as fish reared at 15 °C. On top of increased predation risk, wild larval sDPS green sturgeon were observed to have more empty stomachs at colder temperatures, indicating reduced foraging activity or food availability (Zarri and Palkovacs 2019). Similarly, Poletto et al. (2018) found that combining multiple stressors of cold rearing and restricted feed resulted in lowest relative condition in juvenile nDPS green sturgeon reared to ~ 65 dph. Cold rearing temperatures risk reduced growth, reduced foraging ability, and increased predation. Survival rates and sources of mortality from eggs to early juvenile in wild sDPS green sturgeon should be a priority for future inquiry.

While genetic delineation justifies nDPS and sDPS green sturgeon for separate listing, there is currently no research on whether the underlying genetic differences also lead to differences in thermal physiology. Information on historical temperature regimes in the Rogue and Klamath rivers is limited, but tag and release studies of mature nDPS green sturgeon adults exhibit spawning migrations along similar flow and temperature thresholds, with spring (April–June) upriver migration occurring between 9 and 17 °C and most spawning occurring in temperatures under 18 °C (Erickson and Webb 2007; Benson et al. 2007; Perry et al. 2011). This aligns with optimal nDPS embryo incubation in the laboratory at 15–16 °C and optimal larval yolk-sac depletion at 18–20 °C (Van Eenennaam et al. 2005; Linares-Casenave et al. 2013). When Shasta dam was built on the Sacramento river in 1943, average water temperatures became cooler by about 5℃ in the spring (May and June) and cooler by 7–10 °C in the summer, with very limited evidence suggesting pre-dam average daily temperature at Balls Ferry (18 rkm north of Inks Creek, Fig. 1) around 19 °C in May (California Department of Water Resources 1988; Yates et al. 2008). As such, sDPS green sturgeon likely evolved at similar or slightly warmer temperatures than did nDPS green sturgeon, suggesting the dramatic cold water rearing delays in this study could be a conservative view of sDPS green sturgeon response.

Currently, all early life history sDPS green sturgeon thermal physiology is inferred from nDPS that are reared in a hatchery environment. Significant advances in our knowledge regarding factors affecting phenotypic development in hatchery reared sturgeon (e.g., temperature, hypoxia, salinity, substrate, maternal investment) have been made in recent decades, but the resulting effects of their release on wild populations remain poorly understood (Anderson et al. 2022). Fitness reductions resulting from hatchery rearing are well documented in salmonids (Araki et al. 2008), but in its infancy in sturgeon due to the recent establishment of sturgeon conservation hatchery programs, delayed maturation (8–12 years), and intermittent spawning. In addition to furthering our understanding of how hatchery rearing may affect nDPS green sturgeon, we hope that it will one day be possible to obtain wild sDPS green sturgeon for study to answer these important questions between populations.

### Future management

In most years, when cold water is available, water temperatures are kept artificially cold (< 13.3 °C, U.S. Environmental Protection Agency 2003) in the Sacramento river from April to June to protect incubating and rearing juvenile winter-run Chinook Salmon. The prescribed cold-water releases from Shasta reservoir influence approximately 80 km of the downstream SR, 60 km of which contain adult distribution and confirmed spawning habitat of the sDPS green sturgeon (Fig. 1; Heublein et al. 2009; Poytress et al. 2015). This overlap likely exposes wild sDPS green sturgeon larvae to suboptimal low temperatures during sensitive developmental stages, highlighting a conservation conflict between optimal sDPS green sturgeon and winter-run Chinook salmon management goals.

Given the dramatic stunting in growth due to cold rearing temperatures, we suggest that more suitable spawning and rearing habitat should be created further downstream from the 13.3 °C temperature threshold to improve growth, survival, and recruitment. This would expand spawning habitat to warmer waters and reduce the conflict between managing the same stretch of river for both winter-run Chinook salmon and sDPS green sturgeon. The 2011 confirmation of sDPS green sturgeon spawning in the Feather river for the first time coincided with flow increases and water temperatures rising from 14 to 16 °C, suggesting that green sturgeon consider accessible locations where suitable flow and temperature exist for spawning (Seesholtz et al. 2015). Habitat suitability models may help identify locations where environmental characteristics are similar to preferred spawning habitat with increased flow, appropriate temperatures, or minimal restoration activity. Currently, substrate augmentation via gravel additions is a viable management strategy for salmonid spawning habitat, but such methods have not been investigated for sturgeon in the Sacramento river (Stillwater Sciences 2007). For a range of other sturgeon species, the biggest barrier to effective long-term substrate augmentation is infilling by fine sediments over time, emphasizing the need to collaborate with fluvial geomorphologists to select sites with the appropriate flows and depths for remediation (McAdam et al. 2018). To date, there is a lack of information on egg-to-larva survival, juvenile recruitment, and mortality estimates for all life stages. Our study suggests that artificially suppressed water temperatures may be more detrimental to sDPS green sturgeon than previously thought and confound age estimation of wild-caught fish in Sacramento river spawning habitat.

## Data Availability

The data needed to reproduce the statistical analyses and figures in this study are publicly archived on Figshare at 10.6084/m9.figshare.23713467.
